# MSC-derived extracellular vesicles for spinal cord injury: inflammatory microenvironment modulation, stage-dependent repair mechanisms, and translational challenges

**DOI:** 10.3389/fncel.2026.1960271

**Published:** 2026-09-30

**Authors:** Hang Yu, Yifan Liu, Shulong Yin, Haoyue Hu, Linquan Li, Taotao Zhu, Jie Liu, Sheng Liu, Chenxi Zhao, Shiqing Feng

**Affiliations:** 1 Department of Orthopaedics, Qilu Hospital of Shandong University, Shandong University Centre for Orthopaedics, Advanced Medical Research Institute, Cheeloo College of Medicine, Shandong University, Jinan, Shandong, China; 2 Department of Orthopaedics, The Second Qilu Hospital, Cheeloo College of Medicine, Shandong University, Jinan, Shandong, China; 3 Department of Orthopedics, International Science and Technology Cooperation Base of Spinal Cord Injury, Tianjin Key Laboratory of Spine and Spinal Cord Injury, Tianjin Medical University General Hospital, Tianjin, China

**Keywords:** cell-free therapy, extracellular vesicles, inflammatory microenvironment, microRNA, MSC-EVs, neuroinflammation, spinal cord injury

## Abstract

Spinal cord injury is not only an acute mechanical insult but also the beginning of a prolonged secondary pathological process dominated by inflammatory microenvironmental imbalance. Because the primary traumatic event occurs within minutes and offers a narrow therapeutic window, persistent post-traumatic inflammation, blood-spinal cord barrier disruption, edema, oxidative stress, complement activation, inflammasome signaling, astrocyte reactivity, vascular dysfunction, and extracellular matrix remodeling represent more realistic therapeutic targets. In accordance with the Minimal Information for Studies of Extracellular Vesicles recommendations, this review uses the term MSC-derived extracellular vesicles, abbreviated as MSC-EVs, to describe MSC-derived vesicle preparations reported in the cited studies, including small extracellular vesicles and exosome-like vesicles, unless endosomal biogenesis has been strictly demonstrated. Existing preclinical studies suggest that MSC-EVs may attenuate the inflammatory microenvironment after spinal cord injury by suppressing complement activation, inhibiting NLRP3 inflammasome signaling, modulating heterogeneous microglia/macrophage states, reducing neurotoxic inflammatory astrocyte responses, improving blood-spinal cord barrier integrity, and decreasing inflammatory cytokine production. These anti-inflammatory and barrier-protective effects are closely linked to downstream neuroprotection, angiogenesis, axonal plasticity, remyelination, and tissue repair. Mechanistically, MSC-EVs regulate multiple signaling pathways, including NF-κB, PI3K/Akt, ERK, RhoA/ROCK, PTEN/PI3K/Akt, and Wnt/β-catenin, and deliver regulatory microRNAs such as miR-133b, miR-21, miR-29, miR-126, and miR-486-5p. However, available evidence is uneven: some claims are supported by gain- and loss-of-function experiments, whereas others remain correlative or based on limited animal studies. Beyond microRNAs, MSC-EVs also contain mRNAs, long non-coding RNAs, circular RNAs, tRNA-derived fragments, proteins, and lipids, but their specific roles in spinal cord injury remain less well defined. Reactive astrogliosis is heterogeneous and stage-dependent; A1/A2 terminology may be useful descriptively but should not be treated as a complete classification of astrocyte states. Although MSC-EVs may reduce some risks associated with live-cell transplantation, their clinical translation remains limited by inefficient lesion delivery, biodistribution uncertainty, cargo heterogeneity, storage instability, lack of standardized potency assays, incomplete time-dose–response data, and evolving regulatory requirements. This review summarizes MSC-EVs as modulators of the spinal cord injury inflammatory microenvironment, integrates their actions into an acute-subacute-chronic stage-based model, and discusses the major preclinical and translational barriers that must be addressed before clinical application.

## Introduction

Spinal cord injury refers to structural and/or functional disruption of the spinal cord caused by trauma, disease, or congenital abnormalities, leading to varying degrees of motor, sensory, and autonomic dysfunction below the injury level. Globally, approximately 250,000–500,000 new cases occur annually (Freund et al., 2019; Huang et al., 2020a). Spinal cord injury is frequently accompanied by bladder dysfunction, autonomic disorders, respiratory complications, neuropathic pain, and long-term disability, imposing a substantial burden on patients, families, and society (Liu et al., 2021; Huang et al., 2021).

The pathological process of spinal cord injury involves primary mechanical injury and a prolonged secondary injury cascade. Primary injury refers to tissue tearing, hemorrhage, axonal disruption, and immediate cell death caused by direct mechanical force. Because this process is short-lived, it is difficult to target therapeutically. In contrast, the secondary injury cascade evolves over hours, days, weeks, and even longer periods, and provides a more clinically accessible window for intervention. This cascade is characterized by microenvironmental imbalance, including blood-spinal cord barrier disruption, edema, ischemia, oxidative stress, complement activation, inflammasome signaling, infiltration of peripheral immune cells, activation of resident microglia and astrocytes, release of pro-inflammatory cytokines such as TNF-α and IL-1β, neuronal and oligodendroglial death, and extracellular matrix remodeling (Fan et al., 2018). Therefore, therapeutic strategies for spinal cord injury should focus primarily on the persistent inflammatory microenvironment and secondary tissue destruction initiated by trauma.

Current clinical strategies for spinal cord injury mainly include surgical decompression (Wutte et al., 2019; Fehlings et al., 2012; Yang et al., 2019; Badhiwala et al., 2019), pharmacological interventions (Zarei et al., 2025; Ahmed et al., 2022; Guha and Kumar, 2023), and hemodynamic management (Hawryluk et al., 2015; Ryken et al., 2013; Streijger et al., 2018; Evaniew et al., 2016). These strategies can reduce mechanical compression, limit some secondary damage, or maintain spinal cord perfusion. However, their effects remain limited, particularly in severe and complete injuries (Wutte et al., 2019; Wilson et al., 2017). A major reason is that conventional approaches do not sufficiently reverse the destructive inflammatory microenvironment that sustains neuronal loss, glial dysfunction, vascular leakage, edema, and tissue remodeling after spinal cord injury. Therefore, therapeutic strategies capable of reprogramming the post-injury inflammatory microenvironment may be particularly valuable.

In recent years, mesenchymal stem/stromal cells have attracted attention because of their immunomodulatory and tissue-repair properties. However, live MSC transplantation is limited by concerns regarding immune reactions, vascular obstruction, unstable cell survival, batch variability, ectopic differentiation, and potential tumorigenicity. Increasing evidence indicates that many therapeutic effects of MSCs are mediated through paracrine mechanisms, especially extracellular vesicles and exosome-like vesicles (Nikfarjam et al., 2020; Phinney and Pittenger, 2017; Vizoso et al., 2017; Ferguson et al., 2018; Tkach and Théry, 2016). In this review, these vesicle preparations are collectively referred to as MSC-EVs, unless their endosomal origin has been strictly demonstrated. MSC-EVs carry proteins, lipids, microRNAs, mRNAs, and other regulatory molecules and may modulate immune, neural, glial, and vascular cells in the injured spinal cord (Timmers et al., 2011; Harrell et al., 2019; Xu et al., 2016; Meldolesi, 2018; Wiklander et al., 2015; Kalluri and LeBleu, 2020).

Reactive astrogliosis is one of the most prominent cellular responses after spinal cord injury, and its functions are not uniformly inhibitory. Astrocytes may restrict inflammatory spread, isolate necrotic tissue, regulate ionic and water homeostasis, stabilize lesion boundaries, and participate in blood-spinal cord barrier repair. At the same time, sustained inflammatory phenotypes, excessive extracellular matrix deposition, and fibro-inflammatory tissue remodeling may reduce the permissiveness of the lesion environment. Thus, the therapeutic value of MSC-EVs should be evaluated through their integrated effects on inflammation, glial states, vascular barriers, and extracellular matrix dynamics rather than through a single structural concept.

To distinguish this review from previous broad summaries of MSC-derived vesicles in spinal cord injury, we organize the evidence around three linked questions. First, which components of the post-injury inflammatory microenvironment are directly or indirectly modulated by MSC-EVs? Second, how do vesicle cargos and signaling pathways map onto different pathological stages after injury, including acute inflammation and edema, subacute vascular and glial remodeling, and chronic extracellular matrix remodeling and circuit stabilization? Third, which preclinical variables—MSC source, animal sex, injury model, dose, route, timing, outcome measure, and potency assay—must be standardized before clinical translation? This stage-based and microenvironment-centered framework is intended to synthesize, rather than merely catalogue, the available evidence.

## Search strategy and evidence selection

This article is a narrative review with structured elements of evidence identification and appraisal. Studies were identified through searches of PubMed, Web of Science Core Collection, and Google Scholar. The final search was performed on August 27, 2026. The PubMed search strategy was constructed using combinations of Medical Subject Headings and free-text terms. The core search string was:

(“spinal cord injury” OR “spinal cord injuries” OR SCI) AND (“mesenchymal stem cell” OR “mesenchymal stromal cell” OR MSC OR BMSC OR UCMSC OR ADSC OR “adipose-derived mesenchymal stem cell” OR “umbilical cord mesenchymal stem cell” OR “bone marrow mesenchymal stem cell”) AND (exosome OR exosomes OR “extracellular vesicle” OR “extracellular vesicles” OR “small extracellular vesicle” OR sEV OR “exosome-like vesicle”)

Additional focused searches were performed by combining the above terms with: neuroinflammation, inflammatory microenvironment, microglia, macrophage, astrocyte, A1 astrocyte, A2 astrocyte, blood-spinal cord barrier, complement activation, NLRP3 inflammasome, NF-κB, PI3K/Akt, RhoA/ROCK, PTEN, Wnt/β-catenin, miR-133b, miR-21, miR-29, miR-126, miR-486-5p, long non-coding RNA, circular RNA, tRNA-derived fragment, mRNA, biodistribution, potency assay, GMP, MISEV, and clinical translation.

The search mainly focused on studies published between 2010 and 2026. Earlier publications were included when they provided fundamental concepts regarding extracellular vesicle biology, spinal cord injury pathophysiology, neuroinflammation, animal outcome assessment, or clinical management. Original research articles, reviews, position papers, and guidelines were considered eligible when they were relevant to MSC-derived extracellular vesicles, spinal cord injury, inflammatory regulation, glial responses, vascular repair, RNA cargo, or translational development.

For preclinical animal studies, priority was given to reports that provided information on the animal model, injury severity, MSC source, extracellular vesicle isolation and characterization, dose, route, timing of administration, behavioral outcomes, histological outcomes, and mechanistic assays. Studies using extracellular vesicles derived from non-MSC sources were cited only when they provided mechanistic background relevant to glial or immune regulation. Conference abstracts, non-peer-reviewed reports, and studies lacking essential methodological information were not used as primary evidence for therapeutic efficacy.

## Terminology: exosomes, small extracellular vesicles, and MSC-EVs

The term “exosome” should be used cautiously. According to MISEV recommendations, extracellular vesicles should preferably be described by operational characteristics such as size, density, biochemical markers, or isolation method unless their intracellular origin has been directly demonstrated. Strictly, exosomes are vesicles generated through the endosomal pathway and released after fusion of multivesicular bodies with the plasma membrane. In many spinal cord injury studies, vesicles were isolated by ultracentrifugation, precipitation, size-exclusion chromatography, or commercial kits and were characterized by particle size, morphology, and markers such as CD9, CD63, CD81, TSG101, Alix, or Hsp70. These criteria support identification as small extracellular vesicles or exosome-like vesicles but usually do not prove endosomal origin.

Therefore, in this review, we use MSC-EVs as the preferred umbrella term for MSC-derived extracellular vesicle preparations, including small extracellular vesicles and exosome-like vesicles. When the original studies used “exosomes,” this wording is retained only when necessary to reflect the terminology of the cited papers, but the discussion and interpretation are framed as MSC-EV biology unless endosomal biogenesis has been strictly demonstrated. This distinction is important because heterogeneous extracellular vesicle preparations may include vesicles with different biogenesis routes, cargo profiles, biodistribution patterns, and functional activities. Consistent terminology is also essential for manufacturing, potency testing, regulatory classification, and cross-study comparison.

## MSC-EVs as natural nanotherapeutic carriers: composition, structure, and functional characteristics

### Structural characteristics

MSC-EVs are lipid-bilayer vesicles, and many MSC-EV preparations are enriched in small extracellular vesicles with diameters generally ranging from 30 to 150 nm and densities of approximately 1.13–1.19 g/mL (Lötvall et al., 2014). However, actual MSC-EV preparations may contain heterogeneous vesicle populations depending on the isolation and purification methods used. Under transmission electron microscopy, they often display a cup-shaped morphology due to dehydration during sample preparation, whereas they are generally spherical in a more native state. Their lipid bilayer resembles the cell membrane in composition and organization, allowing MSC-EVs to interact with recipient cells through membrane fusion, receptor-mediated endocytosis, macropinocytosis, phagocytosis, or other uptake mechanisms (Théry et al., 2018).

The membrane of MSC-EVs contains abundant protein complexes and lipid microdomains. Lipid components such as phosphatidylserine, phosphatidylcholine, sphingomyelin, and cholesterol contribute to membrane stability, fluidity, and mechanical strength (Skotland et al., 2017). Proteomic studies have shown that MSC-EVs carry common extracellular vesicle markers such as CD63, CD81, and CD9, molecular chaperones such as Hsp70 and Hsp90, and various adhesion molecules and immunomodulatory proteins (Kowal et al., 2016; Hessvik and Llorente, 2018). These molecules influence vesicle stability, biodistribution, cellular uptake, and biological activity.

The biodistribution of MSC-EVs is influenced by cell source, route of administration, dose, surface molecules, vesicle engineering, and host inflammatory status (Wiklander et al., 2015). After systemic administration, a large proportion of extracellular vesicles may be taken up by the liver, spleen, lung, and mononuclear phagocyte system, while only a fraction reaches the injured spinal cord. Thus, MSC-EVs may show inflammation-associated lesion enrichment in experimental spinal cord injury models, but this should not be equated with fully validated precise targeting (Figure 1).

**Figure 1:**
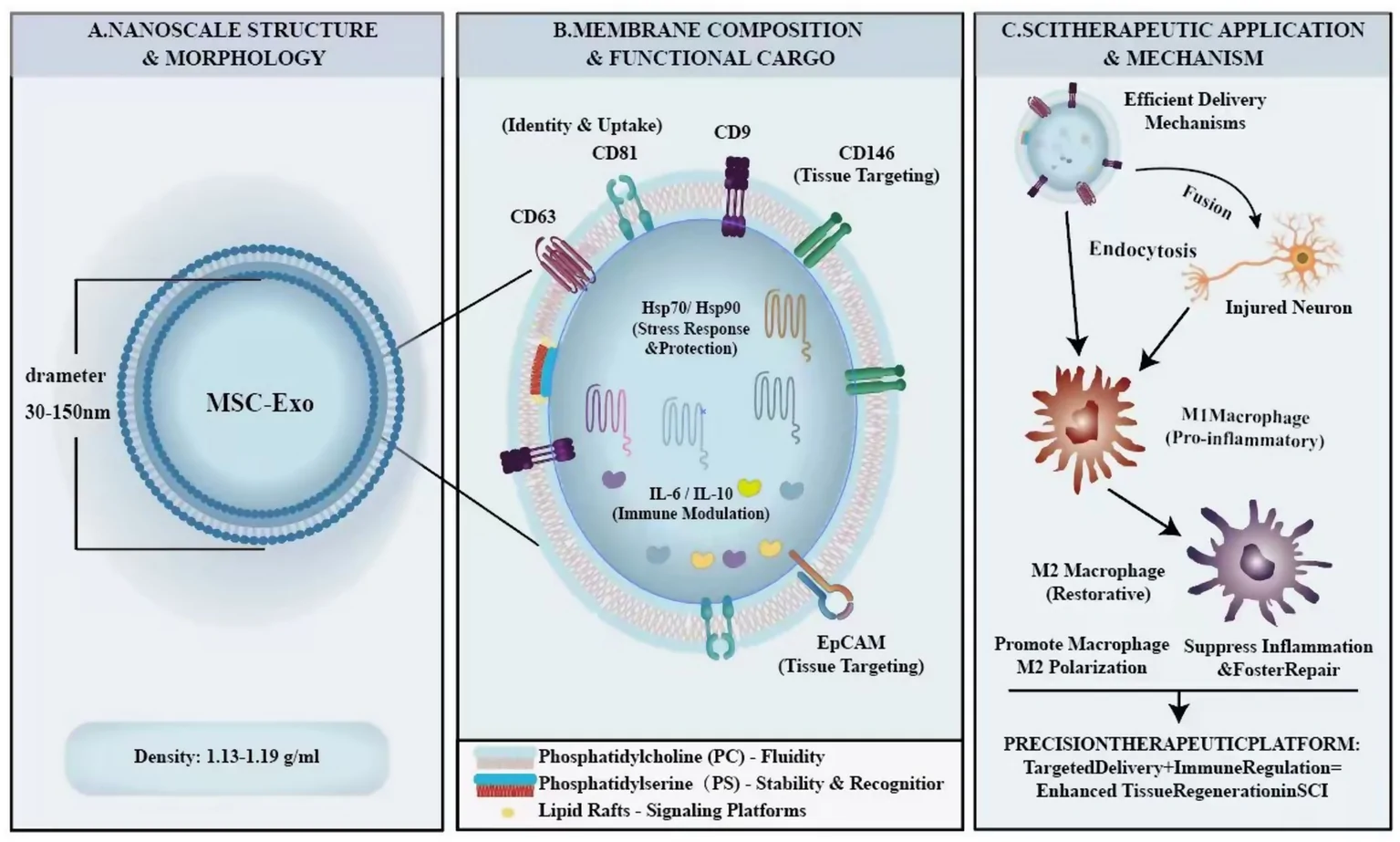
Structure and potential therapeutic mechanisms of MSC-EVs in spinal cord injury. MSC-EVs are nanoscale lipid-bilayer vesicles with a typical diameter of approximately 30–150 nm and a density of approximately 1.13–1.19 g/mL. Their membranes contain phosphatidylserine, phosphatidylcholine, sphingomyelin, cholesterol, tetraspanins such as CD63, CD81, and CD9, heat-shock proteins such as Hsp70 and Hsp90, and other bioactive molecules. These components contribute to vesicle stability, recipient-cell interaction, and cargo transfer. In experimental spinal cord injury models, MSC-EVs may show inflammation-associated lesion enrichment rather than precise targeting, and may be taken up by neural, glial, immune, and vascular cells through endocytosis, membrane fusion, or receptor-mediated mechanisms. Through delivery of proteins, lipids, RNAs, and other regulatory cargos, MSC-EVs may attenuate inflammatory signaling, modulate heterogeneous microglia/macrophage states, reduce neurotoxic inflammatory astrocyte responses, protect the blood-spinal cord barrier, and support tissue repair. Any schematic depiction of macrophage or microglial polarization should be interpreted as a simplified representation of dynamic immune-cell-state modulation rather than as a strict M1/M2 binary classification. These effects remain mainly supported by preclinical evidence and require further validation in human spinal cord injury.

## RNA components

MSC-EVs contain various RNA molecules, including microRNAs, mRNA fragments, tRNA-derived fragments, long non-coding RNAs, and circular RNAs. High-throughput sequencing has shown that MSC-derived extracellular vesicles are enriched in specific microRNA and tRNA species (Baglio et al., 2015; Keam and Hutvagner, 2015; Kumar et al., 2016; Huang et al., 2013b; Li et al., 2015; Rybak-Wolf et al., 2015). These RNA molecules may regulate post-transcriptional gene expression in recipient cells and influence inflammation, apoptosis, angiogenesis, and neural plasticity.

Several microRNAs, including miR-133b, miR-21, miR-29, miR-126, and miR-486-5p, have been implicated in spinal cord injury-related repair mechanisms (Xin et al., 2012; Li et al., 2018; Xu et al., 2019; Sun et al., 2018; Huang et al., 2017; Huang et al., 2020c; Yu et al., 2019; Kang et al., 2019). miR-133b is associated with RhoA/ROCK signaling and axonal plasticity; miR-21 is linked to PTEN/PI3K/Akt, NF-κB, and apoptosis-related pathways; miR-29 may regulate extracellular matrix remodeling; miR-126 is associated with angiogenesis and neuroprotection; and miR-486-5p has been reported to participate in spinal cord injury repair through the PTEN/PI3K/Akt axis (Figure 2).

**Figure 2:**
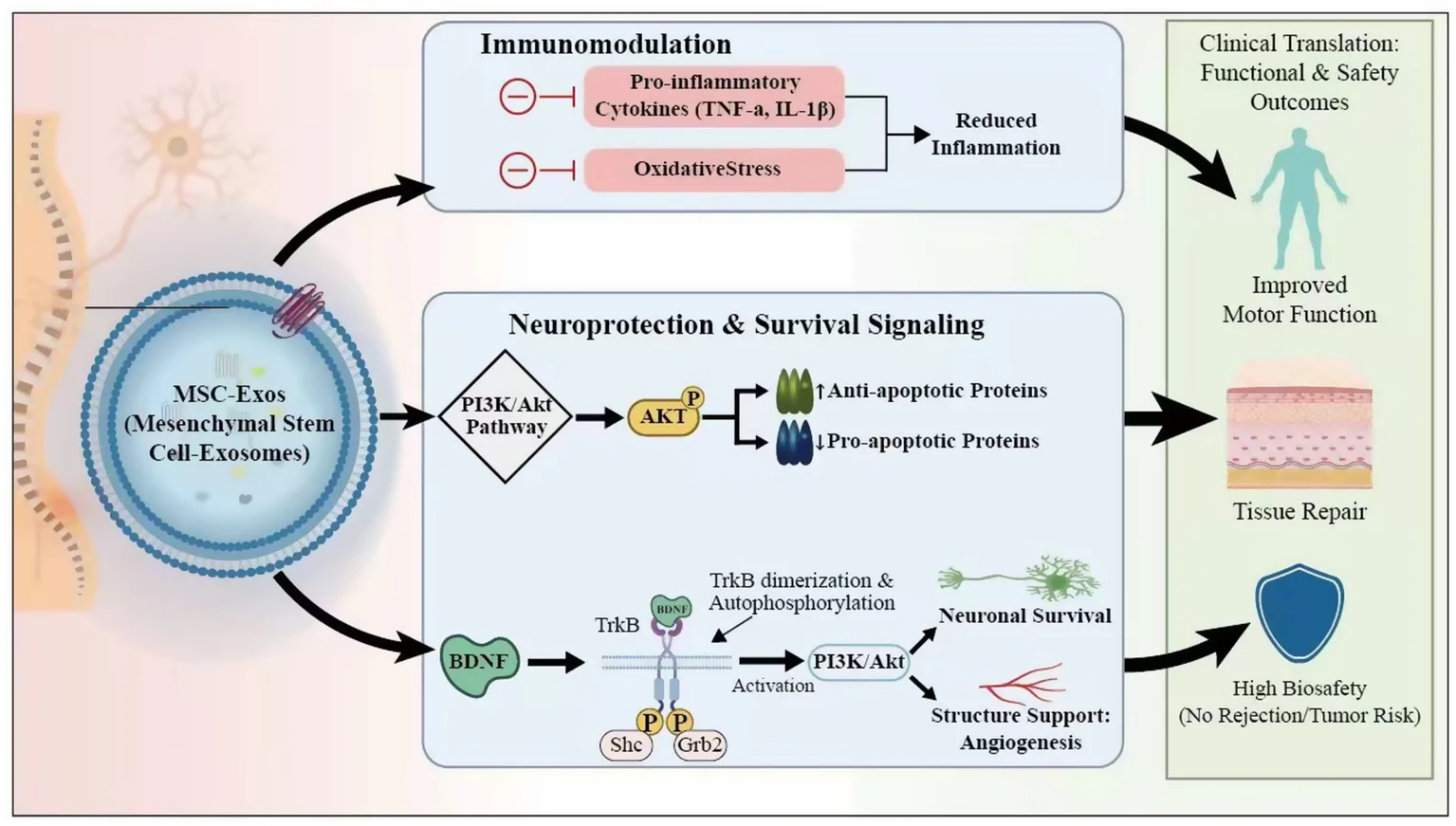
Potential mechanisms by which MSC-EVs support neuroprotection and microenvironmental repair after spinal cord injury. MSC-EVs may regulate several secondary injury mechanisms after spinal cord injury. In experimental models, MSC-EVs have been reported to reduce pro-inflammatory cytokines such as TNF-α and IL-1β, attenuate oxidative stress, and modulate inflammatory signaling pathways. They may also activate survival-related pathways, including PI3K/Akt and ERK signaling, thereby increasing anti-apoptotic responses and reducing apoptosis-associated injury. In addition, MSC-EVs may influence neurotrophic signaling, angiogenesis, blood-spinal cord barrier repair, and glial-cell-state regulation. Functional improvement in animal studies is usually evaluated by behavioral tests such as the BBB locomotor score in rats, the Basso Mouse Scale in mice, inclined-plane testing, ladder-rung or grid-walking tests, footprint analysis, and sometimes electrophysiological recordings. These endpoints should be interpreted together with histological and mechanistic outcomes. Compared with live-cell transplantation, MSC-EVs may have potentially reduced proliferative and embolic risks; however, their long-term clinical safety, biodistribution, optimal dose, and therapeutic efficacy in human spinal cord injury remain to be established.

### Other RNA species beyond microRNAs

Although most spinal cord injury studies of MSC-EVs have focused on microRNAs, vesicles also contain mRNAs, long non-coding RNAs, circular RNAs, tRNA-derived fragments, and other small RNAs. These RNA species may contribute to intercellular communication, but their functional roles in MSC-EV-mediated spinal cord repair remain much less clearly established than those of selected microRNAs.

Vesicle-associated mRNAs may be translated in recipient cells or may reflect the transcriptional state of parental MSCs. In theory, mRNA cargo could influence inflammatory signaling, extracellular matrix remodeling, angiogenesis, or cell-survival pathways in recipient neural, glial, endothelial, and immune cells. However, direct evidence that specific MSC-EV-delivered mRNAs mediate functional recovery after spinal cord injury remains limited. Future studies should combine RNA sequencing, RNase protection assays, vesicle uptake assays, and loss-of-function approaches to distinguish functional mRNA transfer from passive RNA packaging.

Long non-coding RNAs may regulate gene expression by acting as molecular scaffolds, decoys, guides, or competing endogenous RNAs. In spinal cord injury, several lncRNAs have been implicated in inflammation, neuronal apoptosis, astrocyte reactivity, and macrophage/microglial responses, but the contribution of MSC-EV-delivered lncRNAs is still insufficiently defined. Similarly, circular RNAs are enriched and stable in extracellular vesicles and can regulate gene expression through miRNA sponging, RNA-binding protein interaction, or translation of small peptides. Their high stability makes them attractive as vesicle-associated regulatory molecules or biomarkers, but direct mechanistic evidence in MSC-EV-treated spinal cord injury models remains scarce.

tRNA-derived fragments are increasingly recognized as regulatory small RNAs involved in stress responses, translation control, and gene regulation. MSC-derived vesicles can contain tRNA-derived RNA species, but whether these molecules modulate neuroinflammation, glial activation, or vascular repair after spinal cord injury is largely unknown. Thus, the current evidence base is miRNA-dominant, and other RNA species should be considered an important but underexplored direction. Future work should move beyond cargo cataloguing and test whether candidate lncRNAs, circRNAs, mRNAs, or tRNA-derived fragments are necessary and sufficient for specific anti-inflammatory, barrier-protective, or neuroprotective effects.

Therefore, the RNA cargo of MSC-EVs provides an important molecular basis for their anti-inflammatory, neuroprotective, vascular, and tissue-remodeling effects. Nevertheless, extracellular vesicle cargo composition is affected by MSC source, donor status, culture conditions, passage number, oxygen tension, inflammatory priming, and isolation methods. Stable quality-control and potency-assay systems are required before clinical translation (Welsh et al., 2024; Witwer et al., 2019; Lener et al., 2015).

The miRNA-centered emphasis in the mechanistic summary likely reflects not only the biological relevance of selected miRNAs but also a research tendency driven by methodological accessibility. miRNAs are comparatively straightforward to profile and manipulate, and they can often be linked to specific targets using established gain- and loss-of-function approaches. By contrast, proteins, lipids, and non-miRNA RNA species in MSC-EVs require more complex quantitative workflows and cell-type-resolved assays to demonstrate cargo transfer and target engagement. Future studies should therefore adopt integrated multi-omics strategies and prioritize causal validation to determine which cargo classes are necessary and sufficient for defined immunomodulatory, barrier-protective, and neuroprotective effects after SCI.

### MSC-EVs target the inflammatory microenvironment and support neural repair after spinal cord injury

The therapeutic rationale for MSC-EVs in spinal cord injury should be organized around modifiable secondary injury mechanisms rather than the brief primary traumatic event. Among these mechanisms, the persistent inflammatory microenvironment is one of the most important therapeutic targets. After spinal cord injury, activated microglia, infiltrating macrophages, neutrophils, reactive astrocytes, endothelial cells, and meningeal/fibroblast-like cells interact to form a complex inflammatory lesion environment. This environment is characterized by excessive production of TNF-α, IL-1β, IL-6, reactive oxygen species, complement components, and inflammasome-related mediators, which together amplify neuronal apoptosis, oligodendrocyte injury, vascular leakage, edema, and extracellular matrix remodeling (Fan et al., 2018; Liu et al., 2023). Therefore, MSC-EVs should first be considered anti-inflammatory and microenvironment-modulating agents, with neuroprotection, angiogenesis, axonal plasticity, and remyelination representing downstream or parallel repair effects (Khatib et al., 2026; Nakazaki et al., 2026).

### The inflammatory microenvironment as the primary therapeutic target of MSC-EVs

Spinal cord injury initiates a sustained inflammatory microenvironment that is more amenable to therapeutic intervention than the short-lived mechanical insult itself. The injured spinal cord contains activated resident microglia, infiltrating macrophages and neutrophils, reactive astrocytes, damaged endothelial cells, and meningeal/fibroblast-like cells. These cells release pro-inflammatory cytokines, chemokines, reactive oxygen species, complement components, and inflammasome-associated mediators, thereby perpetuating neuronal death, oligodendrocyte loss, demyelination, vascular leakage, edema, and maladaptive tissue remodeling (Fan et al., 2018). This inflammatory microenvironment is not merely a secondary consequence of injury but an active driver of disease progression and functional decline.

Several preclinical studies support the anti-inflammatory role of MSC-EVs in spinal cord injury. Zhao et al. showed that MSC-EVs, termed exosomes in the original study, inhibited complement activation in rats with spinal cord injury, suggesting that MSC-EVs can regulate an important humoral inflammatory pathway after injury (Zhao et al., 2019). Huang et al. reported that systemic administration of MSC-EVs, termed exosomes in the original study, attenuated apoptosis and inflammation while promoting angiogenesis in rat spinal cord injury models (Huang et al., 2017). Epidural fat MSC-derived extracellular vesicles were shown to suppress NLRP3 inflammasome activation and improve functional recovery, further indicating that inflammasome signaling is a relevant target of extracellular vesicle-based therapy (Huang et al., 2020b). MSC-EVs have also been reported to reduce A1-like neurotoxic reactive astrocyte markers through downregulation of phosphorylated NF-κB p65 signaling, linking MSC-EV therapy to modulation of astrocyte-mediated neuroinflammation. Human umbilical cord MSC-derived extracellular vesicles also delivered anti-inflammatory and tissue-remodeling regulatory activities after spinal cord injury. Furthermore, bone marrow MSC-derived extracellular vesicles promoted recovery by improving blood-spinal cord barrier integrity, which may indirectly reduce immune-cell infiltration and inflammatory amplification (Lu et al., 2019).

Although many original studies use M1/M2 terminology to describe macrophage or microglial responses, this binary framework is an oversimplification. After spinal cord injury, resident microglia and infiltrating monocyte-derived macrophages adopt diverse transcriptional and functional states that vary over time and across lesion compartments. Therefore, in this review, M1/M2-related markers such as iNOS, CD86, Arg1, CD206, TNF-α, IL-1β, and IL-10 are discussed as convenient experimental readouts rather than definitive cell identities. Future studies should use multidimensional approaches, including flow cytometry, single-cell transcriptomics, spatial transcriptomics, proteomics, and functional assays, to define how MSC-EVs reshape immune-cell states after spinal cord injury.

These findings indicate that MSC-EVs should not be presented only as carriers of pro-regenerative cargo. Rather, they should be framed primarily as modulators of the post-spinal cord injury inflammatory microenvironment. Their effects on neuronal survival, angiogenesis, axonal plasticity, and tissue repair are closely linked to their capacity to reduce inflammatory toxicity, protect vascular barrier integrity, and regulate glial and immune-cell phenotypes. However, the inflammatory response after spinal cord injury is highly dynamic and cannot be fully described using a simple M1/M2 microglial or macrophage dichotomy. Future studies should define the temporal and spatial effects of MSC-EVs on immune-cell states using standardized inflammatory readouts, such as cytokine profiles, complement activation, inflammasome activity, NF-κB signaling, blood-spinal cord barrier permeability, and single-cell or spatial transcriptomic analyses (Huang et al., 2026).

### MSC-EVs and astrocytic responses

Reactive astrogliosis is an important response of the spinal cord to injury and inflammation. Astrocytes may contribute to restoration of local homeostasis by limiting inflammatory spread, isolating necrotic tissue, regulating extracellular ions and water balance, supporting metabolic homeostasis, and participating in blood-spinal cord barrier repair. Conversely, neurotoxic inflammatory astrocyte phenotypes, abnormal water-channel distribution, and excessive matrix deposition may contribute to edema expansion and impaired repair. Therefore, an effective therapy should reduce maladaptive inflammatory astrocyte phenotypes while preserving beneficial astrocytic functions.

The A1/A2 astrocyte framework has been useful for highlighting that reactive astrocytes can acquire neurotoxic or potentially protective features. A1-like astrocytes are often associated with inflammatory mediators, complement-related genes, and loss of synapse-supportive functions, whereas A2-like astrocytes have been associated with tissue repair, neurotrophic support, and ischemia-related responses. However, similar to the limitations of the M1/M2 microglia/macrophage dichotomy, the A1/A2 classification does not fully capture the diversity, spatial distribution, and temporal evolution of astrocyte states after spinal cord injury. Single-cell and spatial transcriptomic studies increasingly suggest that astrocyte reactivity exists along multiple context-dependent states rather than two fixed phenotypes. Therefore, in this review, the term “A1 neurotoxic reactive astrocytes” is used descriptively when referring to the original studies, but it should not be interpreted as a complete classification of astrocyte biology.

MSC-EVs may contribute to spinal cord injury repair by modulating astrocyte inflammatory states. Liu et al. reported that BMSC-EVs repaired traumatic spinal cord injury by suppressing A1-like neurotoxic reactive astrocyte markers (Liu et al., 2019). Wang et al. further showed that MSC-EVs reduced A1-like astrocyte markers through downregulation of phosphorylated NF-κB p65 in spinal cord injury (Wang et al., 2018). Recent work also suggests that MSC-EVs combined with reactive oxygen species-responsive nanotherapeutics can modulate astrocyte phenotype and target AQP4-related astrocytic edema after spinal cord injury (Cheng et al., 2026). These findings support astrocyte-state modulation as an important component of MSC-EV-mediated inflammatory microenvironment remodeling (Millesi et al., 2026; Zhang et al., 2026a). However, future studies should move beyond A1/A2 marker panels and evaluate astrocyte function directly, including barrier support, glutamate handling, potassium buffering, metabolic coupling, water transport, scar-border formation, and interactions with microglia, endothelial cells, pericytes, and fibroblast-like cells.

Whether MSC-EVs directly promote astrocyte-mediated evacuation of excess edema fluid from swollen perilesional spinal cord tissue requires further study. Because edema contributes to secondary compression, ischemia, and inflammatory amplification, future studies should evaluate astrocytic water transport, AQP4 polarization, vascular permeability, tissue water content, and blood-spinal cord barrier integrity after MSC-EV treatment. Whether MSC-EVs affect astrocytic erythrophagocytosis after hemorrhagic spinal cord injury also remains insufficiently defined. Hemorrhage-derived erythrocytes, hemoglobin, heme, and iron can aggravate oxidative stress and inflammation. Future studies should therefore investigate whether MSC-EVs regulate astrocyte phagocytic activity and hemorrhage-product clearance in the injured spinal cord.

### Neuroprotection and neurotrophic signaling

After spinal cord injury, destructive inflammation, oxidative stress, excitotoxicity, mitochondrial dysfunction, and vascular insufficiency jointly promote neuronal and oligodendroglial death. MSC-EVs may protect neural cells by regulating survival-related signaling pathways, particularly PI3K/Akt and ERK (Zhang et al., 2021; Manning and Toker, 2017; Wang et al., 2024; Zhang et al., 2022). Activation of PI3K/Akt signaling can enhance anti-apoptotic proteins such as Bcl-2 and inhibit pro-apoptotic mediators such as BAD and caspase-9, thereby improving neuronal resistance to secondary injury (Yu et al., 2016).

MSC-EVs may also influence neurotrophic-factor-related signaling. BDNF/TrkB and NGF/TrkA pathways are important regulators of neuronal survival, axonal development, and synaptic plasticity (Cheng et al., 2011; Dahiya et al., 2025; Huang et al., 2011; Huang et al., 2013a; Shi et al., 2024). In the context of spinal cord injury, MSC-EVs may either deliver neurotrophic molecules or indirectly enhance neurotrophic signaling by reducing inflammatory stress and improving the local microenvironment. Recent studies further indicate that MSC-derived extracellular vesicles may participate in neuroprotection and tissue repair through the miR-486-5p/PTEN/PI3K/Akt axis. Thus, the neuroprotective effects of MSC-EVs are best understood as integrated outcomes of inflammatory regulation, cell-survival signaling, and neurotrophic support.

### MicroRNA-mediated modulation of inflammation, survival, and tissue remodeling

MSC-EVs carry multiple microRNAs that may regulate spinal cord injury-related pathological processes. These microRNAs should not be discussed only as pro-regenerative molecules; many of their effects are closely linked to inflammatory microenvironment regulation.

#### miR-133b

miR-133b-enriched MSC-EVs have been reported to promote functional recovery after spinal cord injury (Li et al., 2018). Mechanistically, miR-133b may inhibit RhoA/ROCK signaling, reduce cytoskeletal inhibition, and support axonal plasticity. It may also interact with ERK/CREB, STAT3, and PI3K/Akt/mTOR pathways, thereby contributing to neuronal survival and repair-related gene expression (Li et al., 2018; Xu et al., 2019; Zhang et al., 2019). The beneficial effect of miR-133b should be interpreted as a combined result of neuronal, glial, and inflammatory modulation rather than a purely neuron-intrinsic regeneration mechanism (Figure 3).

**Figure 3:**
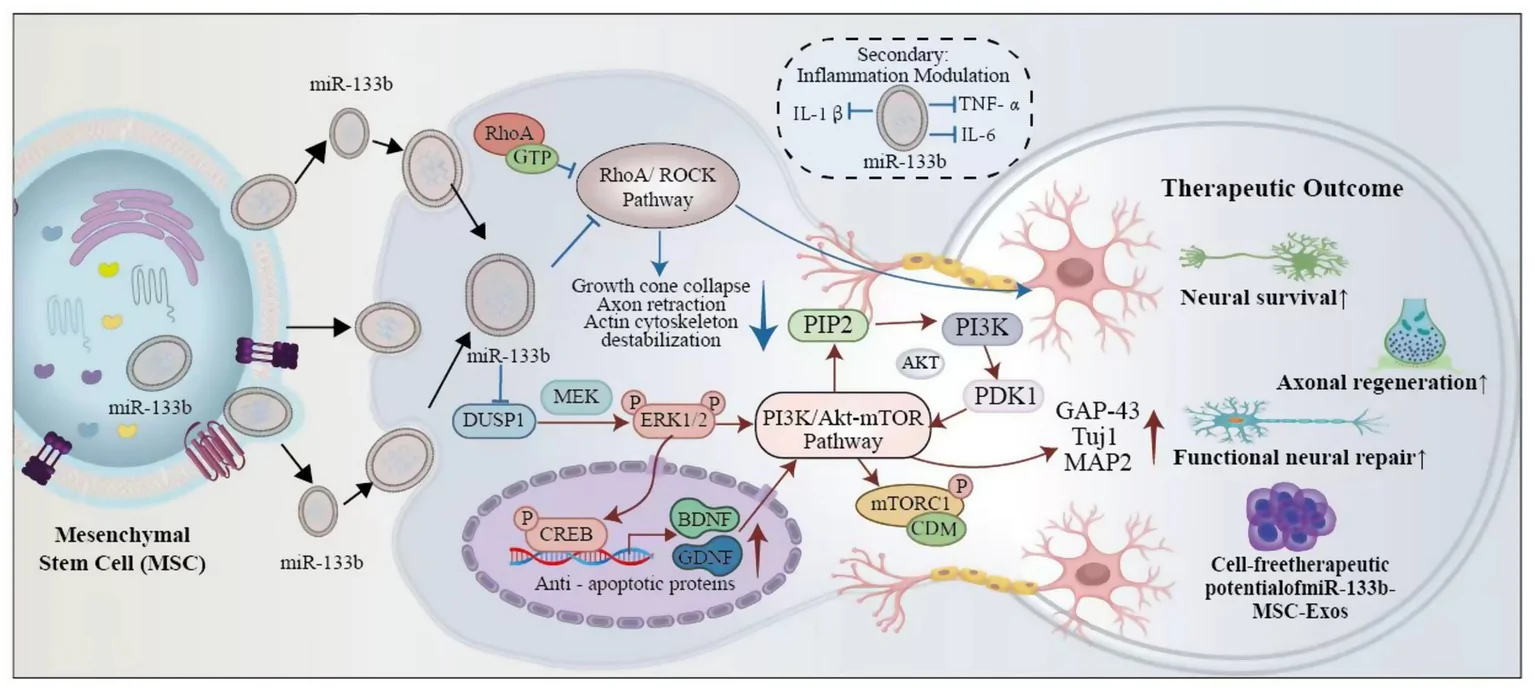
Proposed mechanisms of miR-133b delivered by MSC-EVs in spinal cord injury repair. MSC-EVs enriched with miR-133b may contribute to spinal cord injury repair by regulating multiple molecular pathways. miR-133b has been reported to target RhoA and inhibit RhoA/ROCK signaling, which may reduce cytoskeletal inhibition, growth cone collapse, and axonal retraction. miR-133b may also influence ERK/CREB-related signaling and promote the expression of survival- and plasticity-associated molecules such as Bcl-2, BDNF, GDNF, GAP-43, Tuj1, and MAP2. These effects may contribute to neuronal survival, axonal plasticity, and functional recovery in experimental models. In addition, miR-133b-containing MSC-EVs may indirectly modulate secondary inflammation by regulating inflammatory mediators such as TNF-α and IL-1β. Further studies are needed to define cell-specific targets, dose–response relationships, and long-term safety.

#### miR-21

miR-21 is closely associated with anti-apoptotic and anti-inflammatory regulation. It may target PTEN to activate PI3K/Akt signaling and regulate FasL, PDCD4, and NF-κB-related pathways (Buller et al., 2010; Hu et al., 2013; Kang et al., 2019). In spinal cord injury, miR-21-containing MSC-EVs may reduce neuronal death and modulate inflammatory signaling. miR-21 is context-dependent and may also participate in fibrotic or maladaptive remodeling in certain pathological conditions. Therefore, future studies should define its dose, timing, target-cell specificity, and long-term safety.

#### miR-29

The miR-29 family can regulate extracellular matrix and fibro-inflammatory remodeling. In spinal cord injury models, miR-29b-modified MSC-EVs have been reported to promote repair (Yu et al., 2019). These findings suggest that the miR-29 family may improve the lesion microenvironment by modulating cell-survival, inflammation-related pathways, and local tissue remodeling. However, extracellular matrix regulation is stage-dependent. Excessive or poorly timed suppression of matrix deposition could theoretically interfere with lesion stabilization, vascular support, or scar-border formation. Thus, miR-29-related strategies require careful temporal and cell-specific validation (Figure 4).

**Figure 4:**
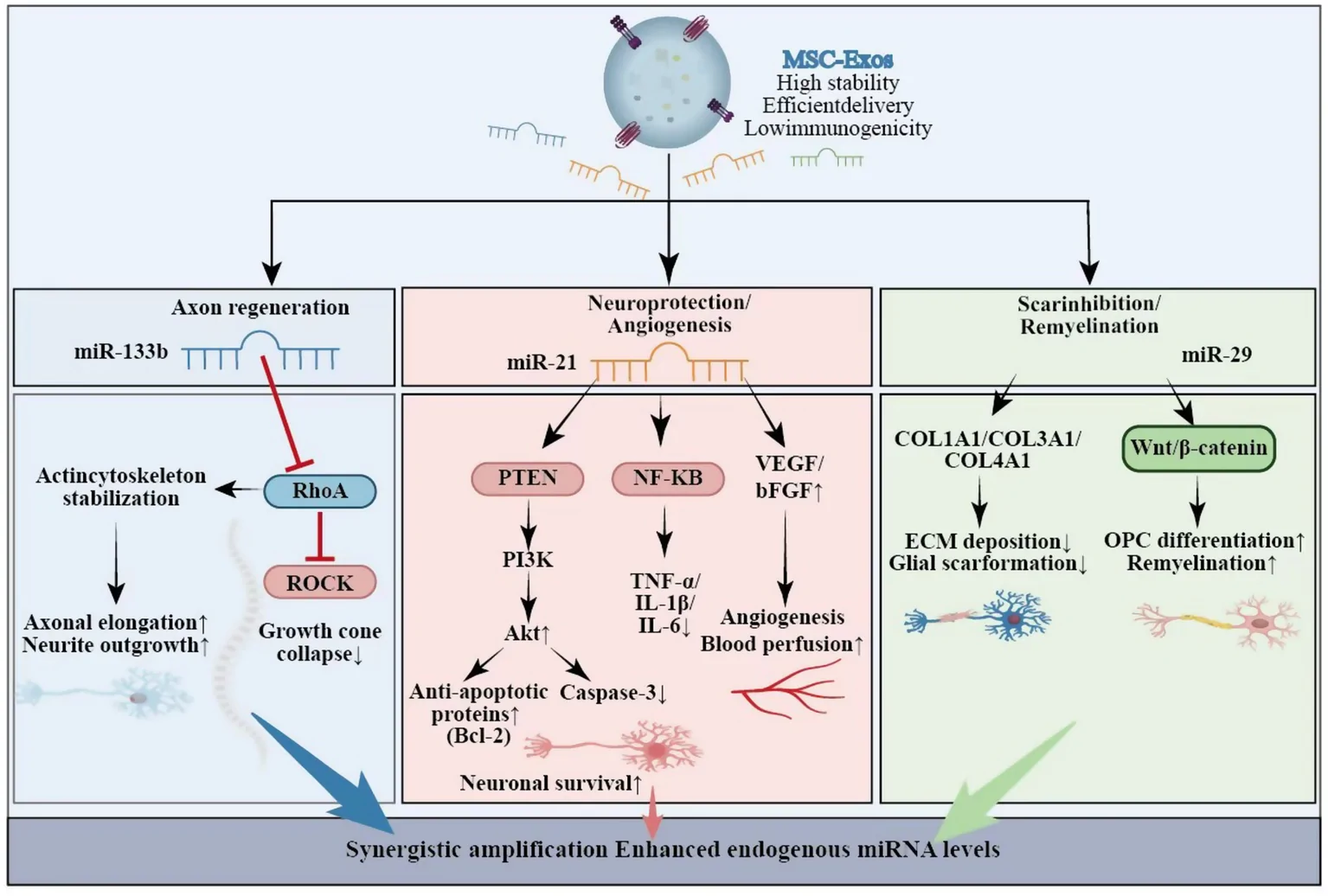
Potential roles of MSC-EVs-delivered miRNAs in regulating inflammation, survival, angiogenesis, and tissue remodeling after spinal cord injury. MSC-EVs can deliver regulatory miRNAs to recipient cells in the injured spinal cord microenvironment. In preclinical studies, miR-133b has been associated with inhibition of RhoA/ROCK signaling and promotion of axonal plasticity; miR-21 may regulate PTEN/PI3K/Akt and NF-κB-related pathways, thereby influencing apoptosis, inflammation, and angiogenesis; and miR-29 may modulate extracellular matrix deposition, fibrotic remodeling, and remyelination-related processes. Other miRNAs, including miR-126 and miR-486-5p, may participate in angiogenesis, neuroprotection, and PTEN/PI3K/Akt signaling. These miRNA-mediated effects should be interpreted as coordinated regulation of inflammation, glial responses, vascular repair, and neural plasticity rather than as a single regenerative mechanism. Their translational value requires standardized vesicle preparation, potency assays, biodistribution studies, and long-term safety evaluation.

#### miR-126 and miR-486-5p

miR-126-modified MSC-EVs promoted angiogenesis and neurogenesis and attenuated apoptosis after spinal cord injury (Huang et al., 2020c). These effects are particularly relevant to inflammatory microenvironment repair because vascular dysfunction and blood-spinal cord barrier disruption amplify immune-cell infiltration and edema. However, improved vascular markers do not necessarily prove durable neural circuit reconstruction. In addition, MSC-EV-delivered miR-486-5p has been reported to alleviate spinal cord injury through the PTEN/PI3K/Akt pathway (Chen et al., 2026). This mechanism is biologically plausible but remains emerging and requires independent replication and loss-of-function validation. Together, these findings support the concept that MSC-EV-derived microRNAs regulate multiple cell populations within the spinal cord injury microenvironment, including neurons, glial cells, vascular cells, and immune cells.

### Strength of evidence and limitations of mechanistic interpretation

Although many preclinical studies report beneficial effects of MSC-EVs after spinal cord injury, the strength of evidence varies considerably across cargos and pathways. Some mechanisms are supported by gain- or loss-of-function experiments, such as the use of miRNA-modified MSCs, miRNA-deficient vesicles, pathway inhibitors, or target-gene validation. Other claims are based mainly on changes in marker expression after vesicle administration and should be interpreted as associative rather than causal.

For example, miR-133b-enriched vesicles have been linked to RhoA/ROCK inhibition and axonal plasticity, but the relative contribution of direct neuronal targeting versus indirect inflammatory or glial modulation remains incompletely resolved. miR-21 may regulate PTEN/PI3K/Akt, FasL, PDCD4, and NF-κB-related pathways, but miR-21 is also context-dependent and may participate in fibrotic or maladaptive remodeling in some disease settings. miR-29 family members may regulate extracellular matrix remodeling, but excessive suppression of matrix components might also interfere with lesion stabilization or repair if applied at inappropriate stages. miR-126 is strongly associated with vascular protection and angiogenesis, but improved vascular markers do not necessarily prove durable neural circuit restoration. miR-486-5p/PTEN/PI3K/Akt signaling is an emerging mechanism, but current spinal cord injury evidence remains limited and requires independent replication.

Negative or neutral findings may be underreported in this field. Many animal studies use small sample sizes, single-dose regimens, short follow-up periods, and male rodents only. Publication bias is therefore a serious concern. Moreover, functional improvement in open-field locomotor scores can be influenced by spontaneous recovery, injury severity variability, animal handling, and observer blinding. Future studies should include prespecified primary outcomes, adequate sample-size calculations, randomized allocation, blinded assessment, both sexes where appropriate, dose–response testing, biodistribution analysis, and predefined criteria for adverse effects. Mechanistic studies should distinguish between cargo enrichment, cargo transfer, target engagement, and functional necessity.

### A stage-based model of MSC-EV actions after spinal cord injury

The biological effects of MSC-EVs should be interpreted in relation to the temporal evolution of spinal cord injury. The post-injury microenvironment changes from acute inflammatory amplification and edema to subacute glial, vascular, and axonal remodeling, and eventually to chronic scar maturation, demyelination, circuit dysfunction, and extracellular matrix stabilization. Therefore, a single cargo or pathway is unlikely to explain therapeutic activity across all stages.

To improve transparency, the stage-based framework in this review is presented with explicit boundaries between empirically supported findings and areas that remain hypothetical. In the available literature, most *in vivo* evidence for MSC-EVs in SCI comes from acute and subacute intervention windows in rodent models, where studies commonly report reductions in inflammatory markers, attenuation of apoptosis, partial preservation of vascular or barrier-associated features, and improvements in early locomotor outcomes. By contrast, evidence in chronic SCI is markedly limited. Proposed chronic-stage actions, including modulation of scar maturation, durable remyelination, long-range circuit reconstruction, and sustained functional improvement, should therefore be regarded as priority directions for future research rather than established therapeutic effects.

During the acute stage, typically within hours to several days after injury, the main therapeutic priorities are suppression of destructive inflammation, stabilization of the blood-spinal cord barrier, reduction of edema, limitation of complement activation, inhibition of inflammasome signaling, and protection of neurons and oligodendrocytes from apoptosis. MSC-EV effects on NF-κB, NLRP3 inflammasome activity, complement activation, PI3K/Akt-mediated survival, and endothelial barrier integrity may be particularly relevant during this period. miR-21 and miR-486-5p may contribute to anti-apoptotic and PTEN/PI3K/Akt-related effects, whereas miR-126 may support early vascular protection and angiogenic signaling.

During the subacute stage, which spans days to weeks after injury, inflammation remains active but tissue repair processes become more prominent. Astrocyte reactivity, microglia/macrophage heterogeneity, angiogenesis, oligodendrocyte survival, axonal sprouting, and extracellular matrix remodeling interact dynamically. miR-133b may be more relevant in this stage because of its association with RhoA/ROCK inhibition and axonal plasticity. miR-126 may support angiogenesis and neurovascular repair, whereas miR-29 family members may begin to influence extracellular matrix deposition and fibro-inflammatory remodeling. At this stage, the therapeutic goal is not simply to suppress inflammation but to shift the lesion environment from destructive inflammation toward controlled remodeling and repair.

During the chronic stage, persistent extracellular matrix deposition, glial and fibrotic scar maturation, demyelination, cystic cavitation, and circuit disconnection become major barriers to recovery. Evidence for MSC-EV therapy in chronic spinal cord injury is much weaker than in acute or subacute models. Potential chronic-stage mechanisms may include modulation of extracellular matrix composition, enhancement of axonal plasticity, remyelination support, and combination with biomaterials, rehabilitation, neuromodulation, or scaffold-based delivery. However, chronic-stage delivery faces additional barriers, including reduced tissue permissiveness, lower inflammatory homing signals, and established anatomical discontinuity. Future studies should therefore separately evaluate acute, subacute, and chronic treatment windows rather than extrapolating from early intervention models.

### Angiogenesis and blood-spinal cord barrier repair

Microvascular injury and blood-spinal cord barrier disruption after spinal cord injury exacerbate hemorrhage, edema, immune-cell infiltration, ischemia, and hypoxia. MSC-EVs may regulate endothelial-cell function by delivering pro-angiogenic microRNAs and proteins. Huang et al. reported that miR-126-modified MSC-EVs promoted angiogenesis and neurogenesis and attenuated apoptosis after spinal cord injury (Huang et al., 2020c). Lu et al. showed that bone marrow MSC-derived extracellular vesicles promoted recovery following spinal cord injury via improvement of blood-spinal cord barrier integrity (Lu et al., 2019). In addition, anti-inflammatory and barrier-protective effects may reinforce each other: reduced inflammation can decrease barrier permeability, whereas barrier repair can limit immune-cell infiltration and inflammatory amplification (Huang et al., 2017).

Engineered delivery systems have further expanded the application of MSC-EVs in spinal cord injury (Zhang et al., 2026b; Yuan et al., 2026). Electroactive hydrogels loaded with human umbilical cord MSC-EVs have been reported to remodel the microenvironment of spinal cord lesion sites and support neural circuit reconstruction (Han et al., 2026). These studies suggest that local delivery and biomaterial-based strategies may enhance EV retention and microenvironmental modulation at the lesion site.

## MSC source-dependent heterogeneity of vesicle cargo and function

MSC-EVs are not a uniform therapeutic entity. Vesicles derived from bone marrow MSCs, adipose-derived MSCs, umbilical cord MSCs, dental pulp MSCs, epidural fat MSCs, or other tissue sources may differ in RNA cargo, protein composition, lipid content, immunomodulatory potency, angiogenic activity, and biodistribution. This source-dependent heterogeneity is highly relevant to spinal cord injury because different pathological stages may require different biological activities. For example, strong anti-inflammatory and barrier-protective activity may be particularly important in the acute stage, whereas angiogenic, axonal-plasticity-related, or extracellular-matrix-modulating effects may be more relevant during subacute and chronic phases.

Bone marrow MSC-derived vesicles are the most frequently studied in spinal cord injury and have been linked to complement inhibition, A1-like astrocyte suppression, autophagy activation, and blood-spinal cord barrier repair. Umbilical cord MSC-derived vesicles are attractive for scalable manufacturing and may possess strong immunomodulatory and pro-angiogenic properties. Adipose-derived MSCs are abundant and accessible, but donor metabolic state may alter vesicle cargo and function, as suggested by studies showing impaired protective effects of vesicles from obese MSCs. Epidural fat MSC-derived vesicles are anatomically relevant to the spinal environment and have been reported to attenuate NLRP3 inflammasome activation. However, direct head-to-head comparisons of MSC sources under identical culture, isolation, dosing, and spinal cord injury conditions are rare. Therefore, claims about superiority of one MSC source over another remain premature.

## Potential advantages and limitations of MSC-EVs compared with cell-based therapies

MSC-EVs may offer several practical and biological advantages over live MSC transplantation, but current evidence does not justify the general conclusion that EVs outperform cell therapies in spinal cord injury. Compared with live cells, MSC-EVs lack nuclei and autonomous proliferative capacity, which may reduce risks related to ectopic engraftment, uncontrolled proliferation, and microvascular obstruction. They may also be easier to store, characterize, and develop as standardized cell-free products (Phinney and Pittenger, 2017; Mendt et al., 2018; Lener et al., 2015; Witwer et al., 2019). In addition, MSC-EVs can deliver multiple bioactive cargos to neurons, glial cells, vascular cells, and immune cells, allowing multi-target modulation of secondary injury after spinal cord injury (Xiao et al., 2026).

However, MSC-EV therapy is not risk-free. After systemic administration, extracellular vesicles may distribute broadly to the liver, spleen, lung, and other organs, raising concerns about off-target effects (Wiklander et al., 2015). Extracellular vesicle cargos are complex and affected by donor characteristics, cell source, culture conditions, and isolation methods, leading to batch variability and difficulty in potency assessment (Witwer et al., 2019). In addition, immunological effects, procoagulant potential, long-term safety, and interactions with host pathological states require systematic evaluation. Therefore, MSC-EVs should be presented as a promising but still investigational therapeutic platform rather than as a clinically proven superior alternative to cell therapy.

## Clinical translation of MSC-EVs for spinal cord injury: current status and challenges

### Current preclinical and translational status

Most evidence supporting MSC-EVs for spinal cord injury remains preclinical. Existing animal studies, mainly using rat spinal cord injury models, suggest that MSC-EVs can improve locomotor recovery, reduce lesion volume, attenuate apoptosis, suppress inflammation, promote angiogenesis, reduce A1-like neurotoxic reactive astrocyte markers, inhibit complement activation, modulate inflammasome signaling, activate autophagy, and improve blood-spinal cord barrier integrity (Zhao et al., 2019; Huang et al., 2017; Huang et al., 2020c; Huang et al., 2020b; Liu et al., 2019; Wang et al., 2018; Ji et al., 2019; Gu et al., 2020; Romanelli et al., 2019; Lu et al., 2019). Recent studies have further extended MSC-EV research to miR-486-5p, AQP4-related astrocytic edema, and engineered hydrogel delivery systems (Cheng et al., 2026; Han et al., 2026). These findings support the potential of MSC-EVs as modulators of the spinal cord injury inflammatory microenvironment and secondary injury cascade.

Functional recovery in rodent spinal cord injury studies is commonly assessed using open-field locomotor scales, particularly the Basso, Beattie, and Bresnahan score for rats and the Basso Mouse Scale for mice, as well as inclined-plane tests, grid-walking or ladder-rung tests, footprint analysis, electrophysiological recordings, and sensory or autonomic assessments when appropriate (Basso et al., 1995; Basso et al., 2006). However, many MSC-EV studies rely primarily on locomotor scores, which may not capture fine motor control, sensory recovery, autonomic function, neuropathic pain, or clinically meaningful circuit reconnection. Therefore, future studies should combine behavioral, electrophysiological, imaging, histological, and molecular endpoints.

Most *in vivo* evidence comes from acute or subacute rat contusion or compression models. These models are useful and reproducible but do not fully represent the heterogeneity of human spinal cord injury, including cervical versus thoracic injuries, complete versus incomplete injuries, penetrating trauma, chronic lesions, age-related comorbidities, and sex-dependent immune responses. Female animals are underrepresented in many preclinical spinal cord injury studies, although sex hormones, immune responses, pain phenotypes, and locomotor recovery may differ between sexes. Chronic-stage MSC-EV delivery has also received far less attention than acute treatment, despite the fact that many patients present after secondary injury has already evolved or live with established chronic injury. Large-animal studies are scarce but important because spinal cord size, lesion volume, cerebrospinal fluid dynamics, immune responses, and delivery constraints differ substantially from those in rodents. Future studies should include both sexes where appropriate, clearly define injury chronicity, report cervical versus thoracic level, and develop large-animal models before clinical translation is attempted.

However, these studies vary substantially in MSC source, extracellular vesicle isolation and characterization methods, dose, timing, route of administration, and outcome measures. Therefore, direct comparison across studies remains difficult. Table 1 summarizes selected preclinical studies of MSC-EVs and engineered EV delivery systems in experimental spinal cord injury and should not be interpreted as evidence of established clinical efficacy in human spinal cord injury.

**Table 1:** Selected preclinical studies of MSC-EVs/sEV-enriched or exosome-like vesicle preparations in experimental spinal cord injury.

Study	EV terminology used in this review/original terminology	MSC source and EV preparation	Species, sex, SCI model and pathological stage	Injury level/severity	EV dose per administration	Injection volume	EV concentration	Route	Timing and dosing schedule	Sample size and follow-up	Functional endpoint and reported effect	Histological/molecular endpoint and reported effect	Mechanistic evidence level	Main proposed mechanism/stage relevance	Reference
Huang et al. (2017)	MSC-EVs; original article used “exosomes”	Rat bone marrow MSC-derived EVs; vesicles characterized by morphology, size distribution and EV markers	Adult rat traumatic SCI model; acute/subacute treatment window	Thoracic traumatic SCI model	100 μg EV protein	200 μL	0.5 μg/μL	Intravenous/systemic administration	Early post-injury systemic administration; repeated injections during early recovery phase	Follow-up to 28 days after SCI	BBB locomotor score significantly improved in MSC-EV-treated rats compared with vehicle-treated SCI rats	Reduced neuronal apoptosis, decreased inflammatory cytokine expression, increased angiogenesis-related markers including endothelial/vessel markers	Therapeutic effect with correlative mechanistic marker analysis; no definitive cargo loss-of-function experiment	Acute anti-inflammatory and anti-apoptotic activity; subacute pro-angiogenic microenvironment repair	Huang et al. (2017)
Liu et al. (2019)	BMSC-EVs; original article used “exosomes”	Bone marrow MSC-derived EVs	Adult rat traumatic SCI model; acute/subacute intervention	Thoracic traumatic SCI	100 μg EV protein	200 μL	0.5 μg/μL	Tail-vein/systemic administration	Early post-injury administration with repeated dosing during the early injury phase	Follow-up to 28 days after SCI	BBB locomotor score improved compared with SCI control animals	Reduced C3-positive/GFAP-positive A1-like neurotoxic reactive astrocyte markers; reduced inflammatory activation around lesion	Mainly correlative astrocyte-state evidence; supports target engagement but not full causal proof	Suppression of neurotoxic inflammatory astrocyte responses; acute-subacute inflammatory microenvironment modulation	Liu et al. (2019)
Wang et al. (2018)	MSC-EVs; original article used “exosomes”	Mesenchymal stem cell-derived EVs	Rat SCI model; acute/subacute treatment	Thoracic SCI model	100 μg EV protein	200 μL	0.5 μg/μL	Tail-vein/systemic administration	Early post-injury administration; repeated administration during early recovery	Follow-up to 28 days after SCI	Locomotor recovery improved compared with SCI vehicle group	Reduced A1-like astrocyte markers including C3; decreased phosphorylated NF-κB p65 in spinal cord tissue	Pathway-associated evidence; mainly marker-based unless inhibitor/rescue experiments are added	NF-κB p65 downregulation linked to reduced inflammatory astrocyte activation; acute-subacute astrocyte-state modulation	Wang et al. (2018)
Zhao et al. (2019)	BMSC-EVs; original article used “exosomes”	Bone marrow MSC-derived EVs	Rat SCI model; acute/subacute treatment	Thoracic SCI model	100 μg EV protein	500 μL	0.2 μg/μL	Tail-vein/systemic administration	Early post-injury administration	Follow-up to 28 days after SCI	BBB score improved compared with SCI control group	Complement activation markers including C3 and C5b-9 were reduced in injured spinal cord tissue	Mechanistic pathway evidence based mainly on complement-marker suppression; causal rescue/blockade evidence limited	Inhibition of complement-mediated inflammatory injury; acute humoral inflammatory regulation	Zhao et al. (2019)
Lu et al. (2019)	BMSC-EVs/extracellular vesicles	Bone marrow MSC-derived extracellular vesicles	Rat SCI model; acute/subacute treatment	Thoracic SCI model	100 μg EV protein	NR	NR	Tail-vein/systemic administration	Early post-injury administration	Follow-up to 28 days after SCI	BBB locomotor score improved compared with SCI vehicle group	Improved blood-spinal cord barrier integrity; reduced Evans blue leakage; preservation of tight-junction proteins including ZO-1, occludin and claudin-5	Correlative barrier-protection evidence; target engagement supported by permeability and tight-junction assays	BSCB stabilization, reduced vascular leakage and secondary inflammatory infiltration; acute-subacute vascular repair	Lu et al. (2019)
Ji et al. (2019)	MSC-EVs; original article used “exosomes”	EVs from normal rat MSCs versus obese rat MSCs; miR-21-related comparison	Rat SCI model; acute/subacute treatment	Thoracic SCI model	100 μg EV protein	NR	NR	Tail-vein/systemic administration	Early post-injury administration	Follow-up to 28 days after SCI	Normal MSC-EVs improved functional recovery more effectively than obese-MSC-EVs	Obese-MSC-EVs showed impaired neuroprotective/anti-inflammatory effects; miR-21 deficiency was associated with reduced therapeutic activity	Comparative and deficiency-associated evidence; stronger than simple correlation but not equivalent to complete rescue validation	Donor metabolic status alters EV miRNA cargo and potency; miR-21 contributes to anti-apoptotic and anti-inflammatory effects	Ji et al. (2019)
Yu et al. (2019)	miR-29b-modified MSC-EVs; original article used “exosomes”	miR-29b-modified MSC-derived EVs	Rat SCI model; acute/subacute treatment	Thoracic SCI model	100 μg EV protein	500 μL	0.2 μg/μL	Tail-vein/systemic administration	Early post-injury administration	Follow-up to 28 days after SCI	BBB locomotor score improved more strongly in miR-29b-modified MSC-EV group than control EV group	Reduced lesion-associated tissue damage and apoptosis; altered extracellular-matrix-related and repair-associated markers	Gain-of-function evidence using miR-29b-modified MSC-EVs; loss-of-function/rescue evidence still needed	miR-29b-associated regulation of apoptosis and extracellular matrix remodeling; subacute remodeling relevance	Yu et al. (2019)
Gu et al. (2020)	BMSC-EVs; original article used “exosomes”	Bone marrow MSC-derived EVs	Rat SCI model; acute/subacute treatment	Thoracic SCI model	100 μg EV protein	200 μL	0.5 μg/μL	Tail-vein/systemic administration	Early post-injury administration	Follow-up to 28 days after SCI	BBB locomotor recovery improved compared with SCI control animals	Increased autophagy-associated markers including LC3-II/LC3-I and Beclin-1; decreased p62; reduced secondary tissue injury	Correlative autophagy evidence unless autophagy inhibitor/rescue experiments are included	Autophagy activation and secondary injury attenuation; acute-subacute cytoprotective mechanism	Gu et al. (2020)
Huang et al. (2020b)	Epidural-fat MSC-EVs; original article used “extracellular vesicles/exosomes”	Epidural fat-derived MSC-EVs	Rat SCI model; acute/subacute treatment	Thoracic SCI model	100 μg EV protein	NR	NR	Tail-vein/systemic administration	Early post-injury administration	Follow-up to 28 days after SCI	BBB locomotor score improved compared with SCI vehicle group	Reduced NLRP3 inflammasome activation; decreased NLRP3, ASC, cleaved caspase-1 and IL-1β expression	Pathway-associated evidence; inflammasome-marker suppression shown, but full causal rescue evidence limited	Suppression of NLRP3 inflammasome-mediated inflammatory amplification; acute inflammatory phase relevance	Huang et al. (2020b)
Huang et al. (2020c)	miR-126-modified MSC-EVs; original article used “exosomes”	miR-126-modified MSC-derived EVs	Rat SCI model; acute/subacute treatment	Thoracic SCI model	100 μg EV protein	NR	NR	Tail-vein/systemic administration	Early post-injury administration	Follow-up to 28 days after SCI	BBB locomotor score improved, with greater recovery in miR-126-modified EV group than unmodified EV group	Increased angiogenesis and neurogenesis markers; reduced apoptosis in lesion/perilesional tissue	Gain-of-function evidence using miR-126-modified MSC-EVs; additional loss-of-function validation would strengthen causality	miR-126-mediated vascular repair, angiogenesis and neuroprotection; acute-subacute neurovascular remodeling	Huang et al. (2020c)

NR, not reported. EV concentration was calculated as EV protein dose divided by injection volume only when both values were explicitly reported in the original article.

Regulatory and clinical statements should be made cautiously. Although extracellular vesicle-based therapeutics are being explored in multiple disease areas, no MSC-EV product has been formally approved by major regulatory agencies for spinal cord injury treatment. Registry-based numbers of extracellular vesicle-related clinical trials change over time and should only be reported with a specific database, search strategy, inclusion criteria, and search date. Similarly, investigational use, emergency use, expanded access, and formal marketing approval should be clearly distinguished. For spinal cord injury, clinical efficacy remains unproven, and translation requires standardized good manufacturing practice-compliant manufacturing, validated identity and purity assays, sterility and endotoxin testing, stability assessment, biodistribution analysis, and mechanism-linked potency assays (Gimona et al., 2017; Welsh et al., 2024; Witwer et al., 2019; Lener et al., 2015).

### Key challenges for clinical translation

#### Inefficient delivery to the injured spinal cord

Systemic administration is convenient but inefficient for lesion delivery. Extracellular vesicle biodistribution is strongly influenced by cell source and administration route, and intravenously delivered vesicles are frequently taken up by the liver, spleen, lung, and mononuclear phagocyte system (Wiklander et al., 2015). Although inflammation may increase local extracellular vesicle accumulation in injured tissue, this should be described as partial lesion enrichment rather than precise targeting. Surface modification, biomaterial-assisted local delivery, intrathecal administration, or engineered targeting strategies may improve lesion delivery, but these approaches require rigorous biodistribution and safety validation (Lai et al., 2014; Richter et al., 2021; Tran et al., 2025).

#### Storage instability and loss of biological activity

Extracellular vesicle stability is affected by storage temperature, freeze–thaw cycles, formulation, and processing methods. Trehalose and other stabilizers may reduce extracellular vesicle aggregation and cryodamage (Bosch et al., 2016), while freeze-drying or pharmaceutical formulation strategies may improve storage feasibility (Mocchi et al., 2021). However, stability should not be assessed only by particle concentration or size distribution. Functional potency assays linked to anti-inflammatory activity, barrier repair, neuroprotection, and glial modulation are required.

#### Donor and batch heterogeneity

MSC-EVs are heterogeneous biological products. Their cargo and function are influenced by MSC tissue source, donor characteristics, passage number, culture conditions, oxygen tension, inflammatory priming, and isolation methods (Witwer et al., 2019; Welsh et al., 2024). This heterogeneity may affect therapeutic reproducibility. Standardized culture systems, bioreactor-based production, defined release criteria, and potency assays are needed to reduce batch-to-batch variability.

#### Incomplete understanding of time-dose–response relationships

The spinal cord injury microenvironment changes dynamically from acute inflammation and edema to subacute tissue remodeling and chronic degeneration. Therefore, MSC-EV therapy is unlikely to have a single optimal mechanism across all stages. Early treatment may need to focus on inflammation, complement activation, edema, and blood-spinal cord barrier disruption; subacute treatment may need to support angiogenesis and axonal plasticity; chronic treatment may need to address extracellular matrix remodeling and circuit stabilization. Future studies should define optimal dosing, timing, and route of administration for each pathological stage.

## Conclusion and future perspective

MSC-EVs represent an investigational cell-free approach for spinal cord injury. Their most coherent therapeutic rationale is modulation of the secondary inflammatory microenvironment rather than direct reversal of the initial mechanical insult. Current preclinical evidence suggests that MSC-EVs can influence complement activation, inflammasome signaling, cytokine production, microglia/macrophage states, astrocyte reactivity, blood-spinal cord barrier integrity, oxidative stress, apoptosis, angiogenesis, axonal plasticity, and extracellular matrix remodeling.

These effects are interconnected and should be interpreted within a stage-dependent framework: acute treatment may primarily target inflammation, edema, and barrier disruption; subacute treatment may support controlled glial and vascular remodeling; and chronic treatment may require combination strategies to overcome established scar architecture and circuit disconnection.

The field now needs more rigorous evidence rather than additional descriptive studies. If MSC-EVs are considered primarily as agents that modulate the inflammatory microenvironment and protect barrier function, preclinical development should place greater emphasis on time-dependent dosing strategies and safety boundaries that are specific to immune regulation. Dose and dosing frequency should be optimized to reduce destructive inflammation in early stages while avoiding excessive or prolonged immunosuppression that could increase infection risk or interfere with repair-associated immune programs. Future studies should therefore provide prespecified dosing rationales, incorporate systemic safety readouts, and evaluate both local spinal inflammatory endpoints and peripheral immune consequences.

This immunomodulator-oriented view also implies that MSC-EVs are more likely to be developed as an adjunctive therapy rather than as a stand-alone intervention. Combination strategies, including biomaterial-assisted local delivery to improve lesion-site exposure, rehabilitation to shape activity-dependent plasticity, and neuromodulation to promote circuit engagement, should be evaluated using mechanistic hypotheses and multidimensional outcome measures beyond open-field locomotion. These outcomes should include electrophysiology, autonomic function, and pain-related endpoints when relevant.

For credible translation, priority should be given to standardized nomenclature and reporting, source-specific comparisons, biodistribution analysis, both-sex animal experiments, chronic-stage models, large-animal validation, and potency assays linked to immunomodulatory and barrier-protective functions. Regulatory translation will require good manufacturing practice-compatible production, validated identity and purity criteria, sterility and endotoxin testing, stability assessment, and clinically relevant safety evaluation. Only after these issues are addressed can MSC-EVs be credibly advanced from experimental microenvironment modulators to candidate therapeutics for human spinal cord injury.
